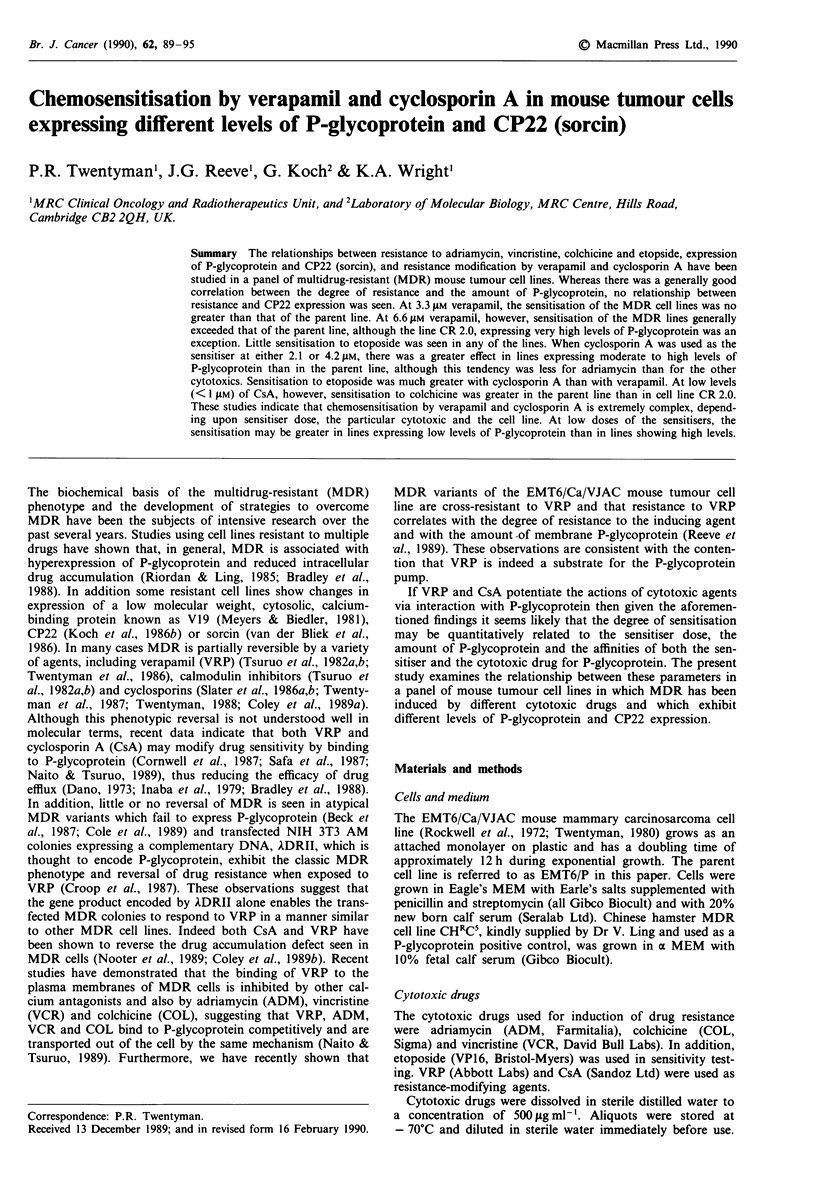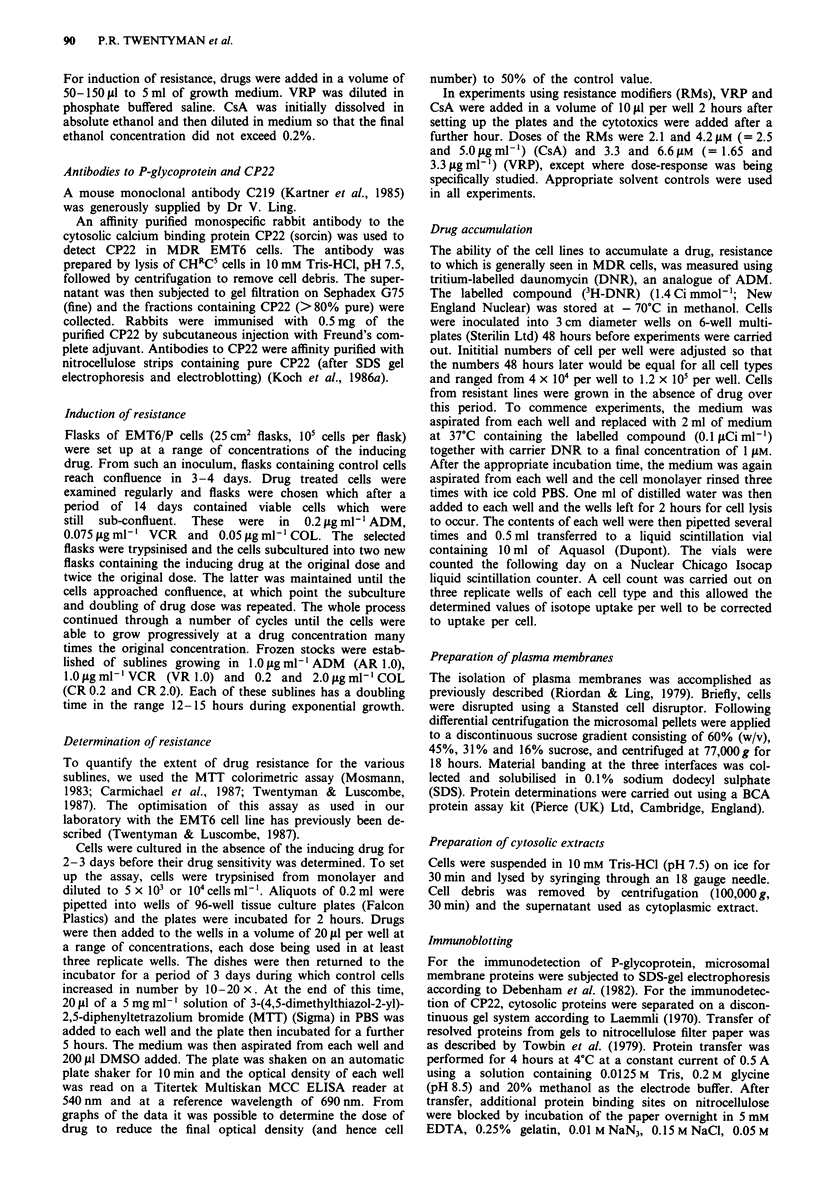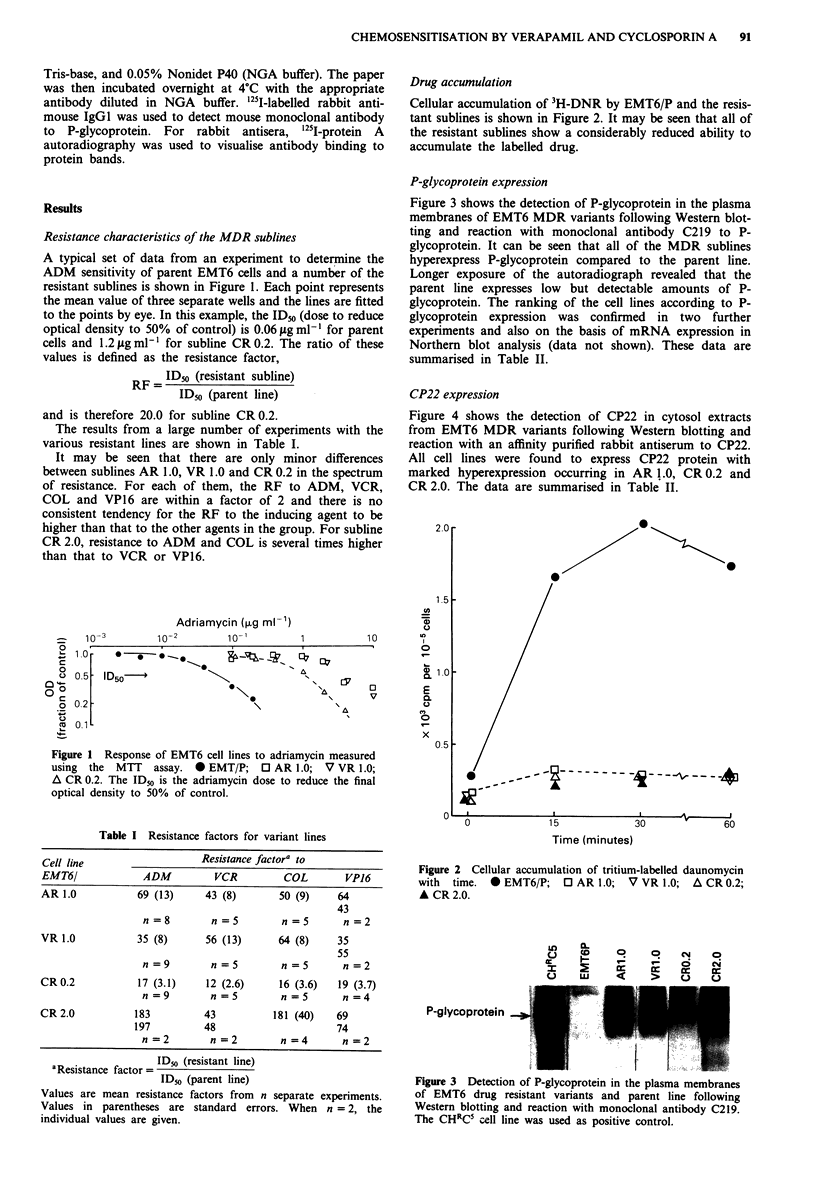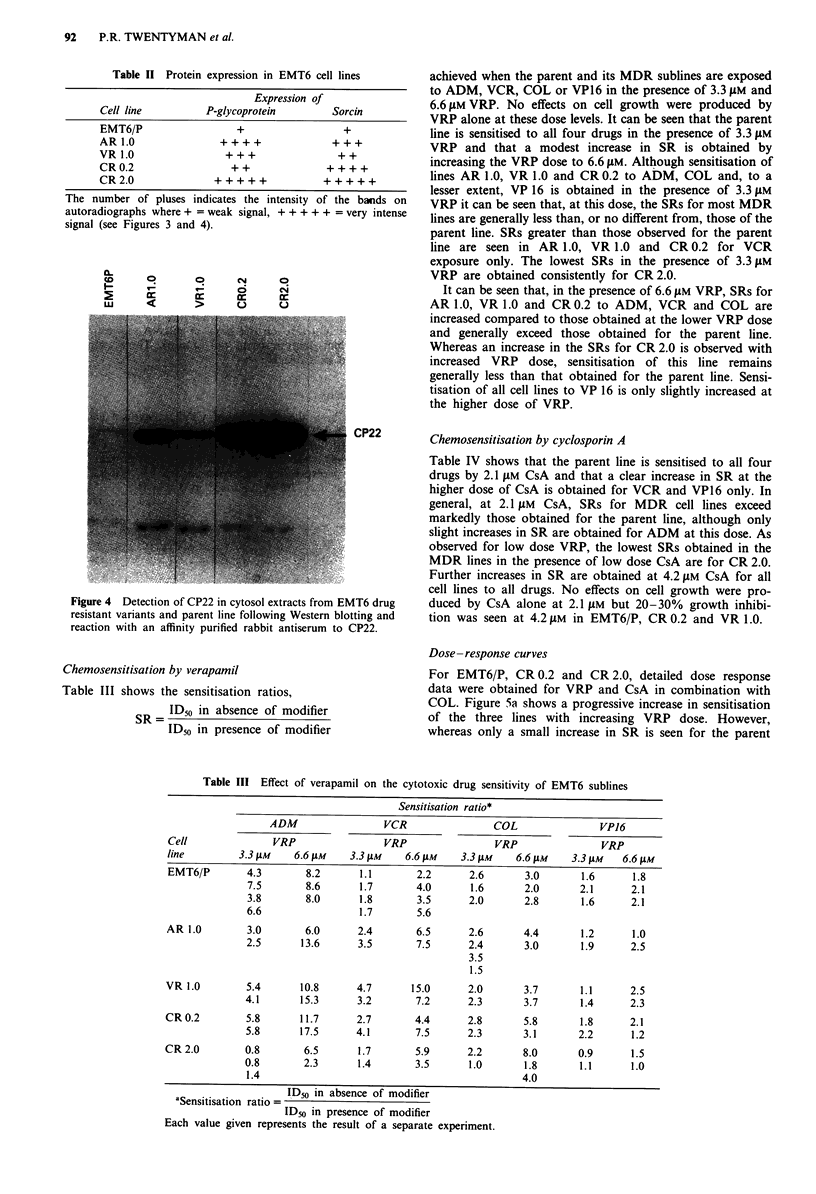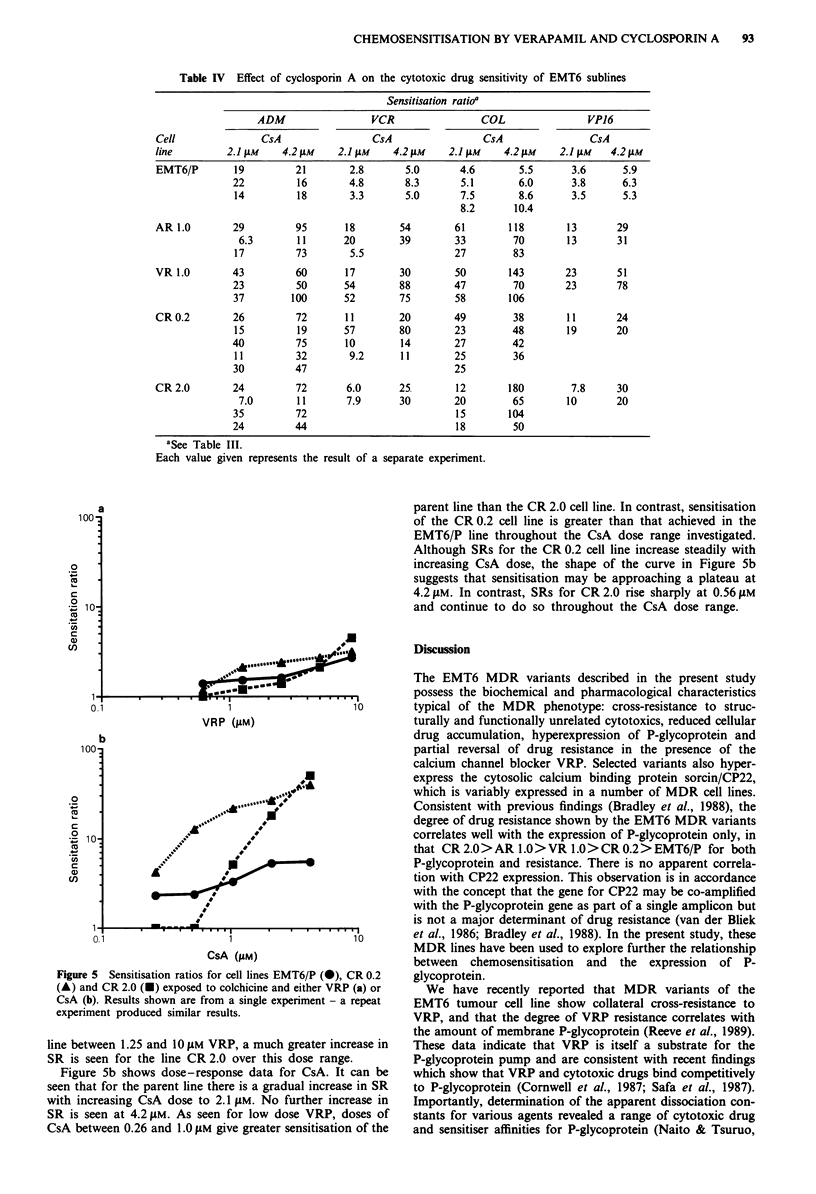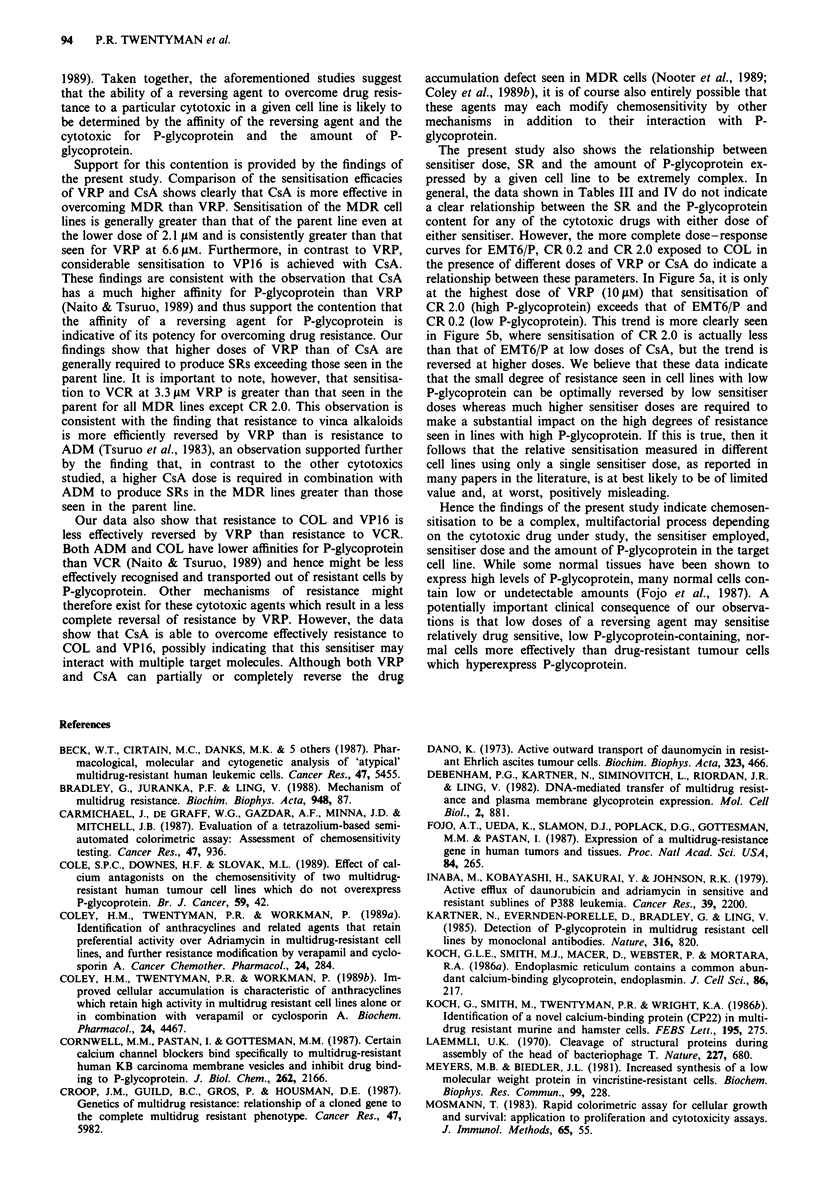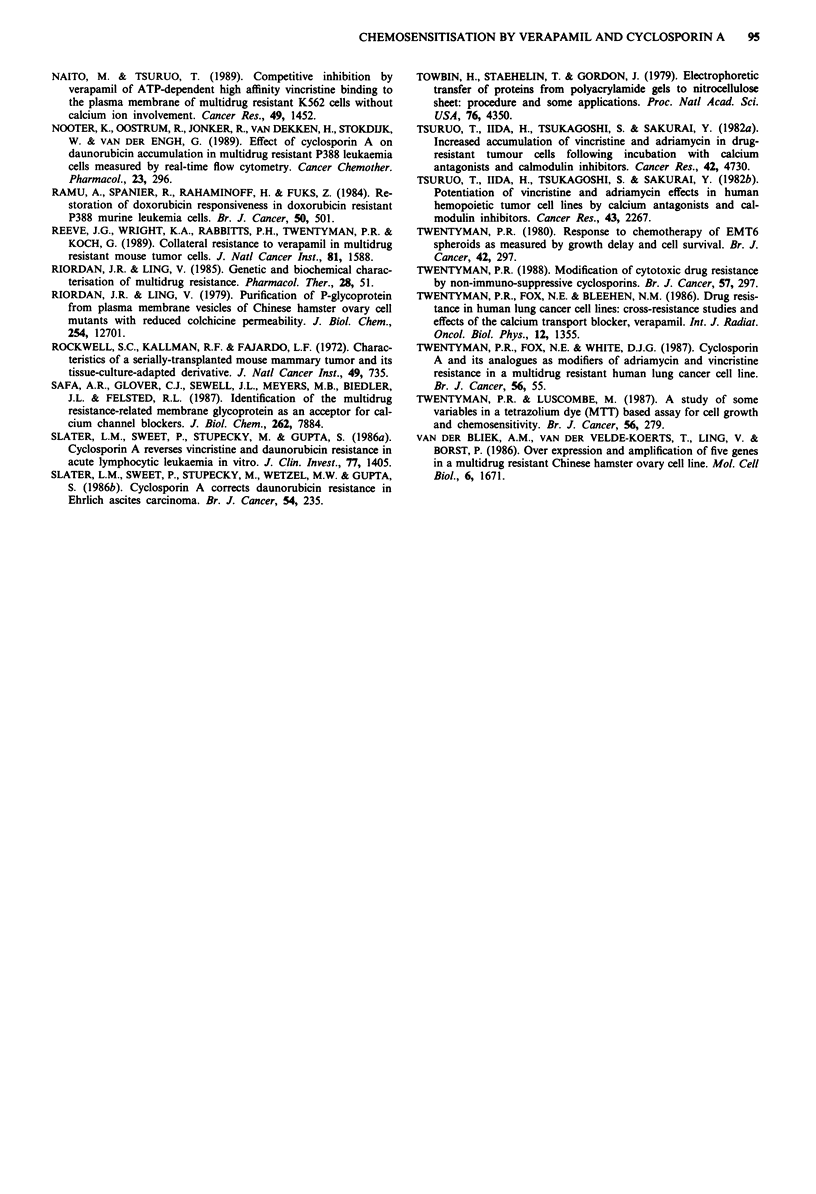# Chemosensitisation by verapamil and cyclosporin A in mouse tumour cells expressing different levels of P-glycoprotein and CP22 (sorcin).

**DOI:** 10.1038/bjc.1990.235

**Published:** 1990-07

**Authors:** P. R. Twentyman, J. G. Reeve, G. Koch, K. A. Wright

**Affiliations:** MRC Clinical Oncology, MRC Centre, Cambridge, UK.

## Abstract

**Images:**


Br. J. Cancer (1990), 62, 89-95 © Macmillan Press Ltd., 1990

### Chemosensitisation by verapamil and cyclosporin A in mouse tumour cells expressing different levels of P-glycoprotein and CP22 (sorcin)

P.R. Twentyman[1], J.G. Reeve[1], G. Koch[2] & K.A. Wright[1]

[1]*MRC Clinical Oncology and Radiotherapeutics Unit, and* [2]*Laboratory of Molecular Biology, MRC Centre, Hills Road, Cambridge CB2 2QH, UK.*

**Summary** The relationships between resistance to adriamycin, vincristine, colchicine and etopside, expression of P-glycoprotein and CP22 (sorcin), and resistance modification by verapamil and cyclosporin A have been studied in a panel of multidrug-resistant (MDR) mouse tumour cell lines. Whereas there was a generally good correlation between the degree of resistance and the amount of P-glycoprotein, no relationship between resistance and CP22 expression was seen. At 3.3 μM verapamil, the sensitisation of the MDR cell lines was no greater than that of the parent line. At 6.6 μM verapamil, however, sensitisation of the MDR lines generally exceeded that of the parent line, although the line CR 2.0, expressing very high levels of P-glycoprotein was an exception. Little sensitisation to etoposide was seen in any of the lines. When cyclosporin A was used as the sensitiser at either 2.1 or 4.2 μM, there was a greater effect in lines expressing moderate to high levels of P-glycoprotein than in the parent line, although this tendency was less for adriamycin than for the other cytotoxics. Sensitisation to etoposide was much greater with cyclosporin A than with verapamil. At low levels (< 1 μM) of CsA, however, sensitisation to colchicine was greater in the parent line than in cell line CR 2.0. These studies indicate that chemosensitisation by verapamil and cyclosporin A is extremely complex, depending upon sensitiser dose, the particular cytotoxic and the cell line. At low doses of the sensitisers, the sensitisation may be greater in lines expressing low levels of P-glycoprotein than in lines showing high levels.

The biochemical basis of the multidrug-resistant (MDR) phenotype and the development of strategies to overcome MDR have been the subjects of intensive research over the past several years. Studies using cell lines resistant to multiple drugs have shown that, in general, MDR is associated with hyperexpression of P-glycoprotein and reduced intracellular drug accumulation (Riordan & Ling, 1985; Bradley *et al.*, 1988). In addition some resistant cell lines show changes in expression of a low molecular weight, cytosolic, calcium-binding protein known as V19 (Meyers & Biedler, 1981), CP22 (Koch *et al.*, 1986*b*) or sorcin (van der Bliek *et al.*, 1986). In many cases MDR is partially reversible by a variety of agents, including verapamil (VRP) (Tsuruo *et al.*, 1982*a,b*; Twentyman *et al.*, 1986), calmodulin inhibitors (Tsuruo *et al.*, 1982*a,b*) and cyclosporins (Slater *et al.*, 1986*a,b*; Twentyman *et al.*, 1987; Twentyman, 1988; Coley *et al.*, 1989*a*). Although this phenotypic reversal is not understood well in molecular terms, recent data indicate that both VRP and cyclosporin A (CsA) may modify drug sensitivity by binding to P-glycoprotein (Cornwell *et al.*, 1987; Safa *et al.*, 1987; Naito & Tsuruo, 1989), thus reducing the efficacy of drug efflux (Dano, 1973; Inaba *et al.*, 1979; Bradley *et al.*, 1988). In addition, little or no reversal of MDR is seen in atypical MDR variants which fail to express P-glycoprotein (Beck *et al.*, 1987; Cole *et al.*, 1989) and transfected NIH 3T3 AM colonies expressing a complementary DNA, λDRII, which is thought to encode P-glycoprotein, exhibit the classic MDR phenotype and reversal of drug resistance when exposed to VRP (Croop *et al.*, 1987). These observations suggest that the gene product encoded by λDRII alone enables the transfected MDR colonies to respond to VRP in a manner similar to other MDR cell lines. Indeed both CsA and VRP have been shown to reverse the drug accumulation defect seen in MDR cells (Nooter *et al.*, 1989; Coley *et al.*, 1989*b*). Recent studies have demonstrated that the binding of VRP to the plasma membranes of MDR cells is inhibited by other calcium antagonists and also by adriamycin (ADM), vincristine (VCR) and colchicine (COL), suggesting that VRP, ADM, VCR and COL bind to P-glycoprotein competitively and are transported out of the cell by the same mechanism (Naito & Tsuruo, 1989). Furthermore, we have recently shown that MDR variants of the EMT6/Ca/VJAC mouse tumour cell line are cross-resistant to VRP and that resistance to VRP correlates with the degree of resistance to the inducing agent and with the amount of membrane P-glycoprotein (Reeve *et al.*, 1989). These observations are consistent with the contention that VRP is indeed a substrate for the P-glycoprotein pump.

If VRP and CsA potentiate the actions of cytotoxic agents via interaction with P-glycoprotein then given the aforementioned findings it seems likely that the degree of sensitisation may be quantitatively related to the sensitiser dose, the amount of P-glycoprotein and the affinities of both the sensitiser and the cytotoxic drug for P-glycoprotein. The present study examines the relationship between these parameters in a panel of mouse tumour cell lines in which MDR has been induced by different cytotoxic drugs and which exhibit different levels of P-glycoprotein and CP22 expression.

### Materials and methods

### *Cells and medium*

The EMT6/Ca/VJAC mouse mammary carcinosarcoma cell line (Rockwell *et al.*, 1972; Twentyman, 1980) grows as an attached monolayer on plastic and has a doubling time of approximately 12 h during exponential growth. The parent cell line is referred to as EMT6/P in this paper. Cells were grown in Eagle's MEM with Earle's salts supplemented with penicillin and streptomycin (all Gibco Biocult) and with 20% new born calf serum (Seralab Ltd). Chinese hamster MDR cell line $CH^RC^5$, kindly supplied by Dr V. Ling and used as a P-glycoprotein positive control, was grown in α MEM with 10% fetal calf serum (Gibco Biocult).

### *Cytotoxic drugs*

The cytotoxic drugs used for induction of drug resistance were adriamycin (ADM, Farmitalia), colchicine (COL, Sigma) and vincristine (VCR, David Bull Labs). In addition, etoposide (VP16, Bristol-Myers) was used in sensitivity testing. VRP (Abbott Labs) and CsA (Sandoz Ltd) were used as resistance-modifying agents.

Cytotoxic drugs were dissolved in sterile distilled water to a concentration of 500 μg $ml^{-1}$. Aliquots were stored at − 70°C and diluted in sterile water immediately before use.

Correspondence: P.R. Twentyman.
Received 13 December 1989; and in revised form 16 February 1990.

For induction of resistance, drugs were added in a volume of 50–150 µl to 5 ml of growth medium. VRP was diluted in phosphate buffered saline. CsA was initially dissolved in absolute ethanol and then diluted in medium so that the final ethanol concentration did not exceed 0.2%.

*Antibodies to P-glycoprotein and CP22*

A mouse monoclonal antibody C219 (Kartner et al., 1985) was generously supplied by Dr V. Ling.

An affinity purified monospecific rabbit antibody to the cytosolic calcium binding protein CP22 (sorcin) was used to detect CP22 in MDR EMT6 cells. The antibody was prepared by lysis of $CH^RC^5$ cells in 10 mM Tris-HCl, pH 7.5, followed by centrifugation to remove cell debris. The supernatant was then subjected to gel filtration on Sephadex G75 (fine) and the fractions containing CP22 (>80% pure) were collected. Rabbits were immunised with 0.5 mg of the purified CP22 by subcutaneous injection with Freund's complete adjuvant. Antibodies to CP22 were affinity purified with nitrocellulose strips containing pure CP22 (after SDS gel electrophoresis and electroblotting) (Koch et al., 1986a).

*Induction of resistance*

Flasks of EMT6/P cells (25 $cm^2$ flasks, $10^5$ cells per flask) were set up at a range of concentrations of the inducing drug. From such an inoculum, flasks containing control cells reach confluence in 3–4 days. Drug treated cells were examined regularly and flasks were chosen which after a period of 14 days contained viable cells which were still sub-confluent. These were in 0.2 $\mu g\ ml^{-1}$ ADM, 0.075 $\mu g\ ml^{-1}$ VCR and 0.05 $\mu g\ ml^{-1}$ COL. The selected flasks were trypsinised and the cells subcultured into two new flasks containing the inducing drug at the original dose and twice the original dose. The latter was maintained until the cells approached confluence, at which point the subculture and doubling of drug dose was repeated. The whole process continued through a number of cycles until the cells were able to grow progressively at a drug concentration many times the original concentration. Frozen stocks were established of sublines growing in 1.0 $\mu g\ ml^{-1}$ ADM (AR 1.0), 1.0 $\mu g\ ml^{-1}$ VCR (VR 1.0) and 0.2 and 2.0 $\mu g\ ml^{-1}$ COL (CR 0.2 and CR 2.0). Each of these sublines has a doubling time in the range 12–15 hours during exponential growth.

*Determination of resistance*

To quantify the extent of drug resistance for the various sublines, we used the MTT colorimetric assay (Mosmann, 1983; Carmichael et al., 1987; Twentyman & Luscombe, 1987). The optimisation of this assay as used in our laboratory with the EMT6 cell line has previously been described (Twentyman & Luscombe, 1987).

Cells were cultured in the absence of the inducing drug for 2–3 days before their drug sensitivity was determined. To set up the assay, cells were trypsinised from monolayer and diluted to $5 \times 10^3$ or $10^4$ cells $ml^{-1}$. Aliquots of 0.2 ml were pipetted into wells of 96-well tissue culture plates (Falcon Plastics) and the plates were incubated for 2 hours. Drugs were then added to the wells in a volume of 20 µl per well at a range of concentrations, each dose being used in at least three replicate wells. The dishes were then returned to the incubator for a period of 3 days during which control cells increased in number by 10–20 ×. At the end of this time, 20 µl of a 5 $mg\ ml^{-1}$ solution of 3-(4,5-dimethylthiazol-2-yl)-2,5-diphenyltetrazolium bromide (MTT) (Sigma) in PBS was added to each well and the plate then incubated for a further 5 hours. The medium was then aspirated from each well and 200 µl DMSO added. The plate was shaken on an automatic plate shaker for 10 min and the optical density of each well was read on a Titertek Multiskan MCC ELISA reader at 540 nm and at a reference wavelength of 690 nm. From graphs of the data it was possible to determine the dose of drug to reduce the final optical density (and hence cell number) to 50% of the control value.

In experiments using resistance modifiers (RMs), VRP and CsA were added in a volume of 10 µl per well 2 hours after setting up the plates and the cytotoxics were added after a further hour. Doses of the RMs were 2.1 and 4.2 µM (= 2.5 and 5.0 $\mu g\ ml^{-1}$) (CsA) and 3.3 and 6.6 µM (= 1.65 and 3.3 $\mu g\ ml^{-1}$) (VRP), except where dose-response was being specifically studied. Appropriate solvent controls were used in all experiments.

*Drug accumulation*

The ability of the cell lines to accumulate a drug, resistance to which is generally seen in MDR cells, was measured using tritium-labelled daunomycin (DNR), an analogue of ADM. The labelled compound ($^3$H-DNR) (1.4 Ci $mmol^{-1}$; New England Nuclear) was stored at − 70°C in methanol. Cells were inoculated into 3 cm diameter wells on 6-well multiplates (Sterilin Ltd) 48 hours before experiments were carried out. Initital numbers of cell per well were adjusted so that the numbers 48 hours later would be equal for all cell types and ranged from $4 \times 10^4$ per well to $1.2 \times 10^5$ per well. Cells from resistant lines were grown in the absence of drug over this period. To commence experiments, the medium was aspirated from each well and replaced with 2 ml of medium at 37°C containing the labelled compound (0.1 $\mu Ci\ ml^{-1}$) together with carrier DNR to a final concentration of 1 µM. After the appropriate incubation time, the medium was again aspirated from each well and the cell monolayer rinsed three times with ice cold PBS. One ml of distilled water was then added to each well and the wells left for 2 hours for cell lysis to occur. The contents of each well were then pipetted several times and 0.5 ml transferred to a liquid scintillation vial containing 10 ml of Aquasol (Dupont). The vials were counted the following day on a Nuclear Chicago Isocap liquid scintillation counter. A cell count was carried out on three replicate wells of each cell type and this allowed the determined values of isotope uptake per well to be corrected to uptake per cell.

*Preparation of plasma membranes*

The isolation of plasma membranes was accomplished as previously described (Riordan & Ling, 1979). Briefly, cells were disrupted using a Stansted cell disruptor. Following differential centrifugation the microsomal pellets were applied to a discontinuous sucrose gradient consisting of 60% (w/v), 45%, 31% and 16% sucrose, and centrifuged at 77,000 g for 18 hours. Material banding at the three interfaces was collected and solubilised in 0.1% sodium dodecyl sulphate (SDS). Protein determinations were carried out using a BCA protein assay kit (Pierce (UK) Ltd, Cambridge, England).

*Preparation of cytosolic extracts*

Cells were suspended in 10 mM Tris-HCl (pH 7.5) on ice for 30 min and lysed by syringing through an 18 gauge needle. Cell debris was removed by centrifugation (100,000 g, 30 min) and the supernatant used as cytoplasmic extract.

*Immunoblotting*

For the immunodetection of P-glycoprotein, microsomal membrane proteins were subjected to SDS-gel electrophoresis according to Debenham et al. (1982). For the immunodetection of CP22, cytosolic proteins were separated on a discontinuous gel system according to Laemmli (1970). Transfer of resolved proteins from gels to nitrocellulose filter paper was as described by Towbin et al. (1979). Protein transfer was performed for 4 hours at 4°C at a constant current of 0.5 A using a solution containing 0.0125 M Tris, 0.2 M glycine (pH 8.5) and 20% methanol as the electrode buffer. After transfer, additional protein binding sites on nitrocellulose were blocked by incubation of the paper overnight in 5 mM EDTA, 0.25% gelatin, 0.01 M $NaN_3$, 0.15 M NaCl, 0.05 M

Tris-base, and 0.05% Nonidet P40 (NGA buffer). The paper was then incubated overnight at 4°C with the appropriate antibody diluted in NGA buffer. $^{125}$I-labelled rabbit anti-mouse IgG1 was used to detect mouse monoclonal antibody to P-glycoprotein. For rabbit antisera, $^{125}$I-protein A autoradiography was used to visualise antibody binding to protein bands.

### Results

### *Resistance characteristics of the MDR sublines*

A typical set of data from an experiment to determine the ADM sensitivity of parent EMT6 cells and a number of the resistant sublines is shown in Figure 1. Each point represents the mean value of three separate wells and the lines are fitted to the points by eye. In this example, the $ID_{50}$ (dose to reduce optical density to 50% of control) is 0.06 μg ml$^{-1}$ for parent cells and 1.2 μg ml$^{-1}$ for subline CR 0.2. The ratio of these values is defined as the resistance factor,

$$RF = \frac{ID_{50}\text{ (resistant subline)}}{ID_{50}\text{ (parent line)}}$$

and is therefore 20.0 for subline CR 0.2.

The results from a large number of experiments with the various resistant lines are shown in Table I.

It may be seen that there are only minor differences between sublines AR 1.0, VR 1.0 and CR 0.2 in the spectrum of resistance. For each of them, the RF to ADM, VCR, COL and VP16 are within a factor of 2 and there is no consistent tendency for the RF to the inducing agent to be higher than that to the other agents in the group. For subline CR 2.0, resistance to ADM and COL is several times higher than that to VCR or VP16.

**Figure 1** Response of EMT6 cell lines to adriamycin measured using the MTT assay. ● EMT/P; □ AR 1.0; ∇ VR 1.0; Δ CR 0.2. The $ID_{50}$ is the adriamycin dose to reduce the final optical density to 50% of control.

**Table I** Resistance factors for variant lines

| *Cell line EMT6/* | *Resistance factor$^a$ to* | | | |
|---|---|---|---|---|
| | *ADM* | *VCR* | *COL* | *VP16* |
| AR 1.0 | 69 (13) | 43 (8) | 50 (9) | 64 |
| | | | | 43 |
| | $n = 8$ | $n = 5$ | $n = 5$ | $n = 2$ |
| VR 1.0 | 35 (8) | 56 (13) | 64 (8) | 35 |
| | | | | 55 |
| | $n = 9$ | $n = 5$ | $n = 5$ | $n = 2$ |
| CR 0.2 | 17 (3.1) | 12 (2.6) | 16 (3.6) | 19 (3.7) |
| | $n = 9$ | $n = 5$ | $n = 5$ | $n = 4$ |
| CR 2.0 | 183 | 43 | 181 (40) | 69 |
| | 197 | 48 | | 74 |
| | $n = 2$ | $n = 2$ | $n = 4$ | $n = 2$ |

$^a$Resistance factor $= \frac{ID_{50}\text{ (resistant line)}}{ID_{50}\text{ (parent line)}}$

Values are mean resistance factors from $n$ separate experiments. Values in parentheses are standard errors. When $n = 2$, the individual values are given.

### *Drug accumulation*

Cellular accumulation of $^3$H-DNR by EMT6/P and the resistant sublines is shown in Figure 2. It may be seen that all of the resistant sublines show a considerably reduced ability to accumulate the labelled drug.

### *P-glycoprotein expression*

Figure 3 shows the detection of P-glycoprotein in the plasma membranes of EMT6 MDR variants following Western blotting and reaction with monoclonal antibody C219 to P-glycoprotein. It can be seen that all of the MDR sublines hyperexpress P-glycoprotein compared to the parent line. Longer exposure of the autoradiograph revealed that the parent line expresses low but detectable amounts of P-glycoprotein. The ranking of the cell lines according to P-glycoprotein expression was confirmed in two further experiments and also on the basis of mRNA expression in Northern blot analysis (data not shown). These data are summarised in Table II.

### *CP22 expression*

Figure 4 shows the detection of CP22 in cytosol extracts from EMT6 MDR variants following Western blotting and reaction with an affinity purified rabbit antiserum to CP22. All cell lines were found to express CP22 protein with marked hyperexpression occurring in AR 1.0, CR 0.2 and CR 2.0. The data are summarised in Table II.

**Figure 2** Cellular accumulation of tritium-labelled daunomycin with time. ● EMT6/P; □ AR 1.0; ∇ VR 1.0; Δ CR 0.2; ▲ CR 2.0.

**Figure 3** Detection of P-glycoprotein in the plasma membranes of EMT6 drug resistant variants and parent line following Western blotting and reaction with monoclonal antibody C219. The CH$^R$C$^5$ cell line was used as positive control.

**Table II** Protein expression in EMT6 cell lines

| *Cell line* | *Expression of* *P-glycoprotein* | *Sorcin* |
|---|---|---|
| EMT6/P | + | + |
| AR 1.0 | ++++ | +++ |
| VR 1.0 | +++ | ++ |
| CR 0.2 | ++ | ++++ |
| CR 2.0 | +++++ | +++++ |

The number of pluses indicates the intensity of the bands on autoradiographs where + = weak signal, +++++ = very intense signal (see Figures 3 and 4).

**Figure 4** Detection of CP22 in cytosol extracts from EMT6 drug resistant variants and parent line following Western blotting and reaction with an affinity purified rabbit antiserum to CP22.

*Chemosensitisation by verapamil*

Table III shows the sensitisation ratios,

$$SR = \frac{ID_{50} \text{ in absence of modifier}}{ID_{50} \text{ in presence of modifier}}$$

achieved when the parent and its MDR sublines are exposed to ADM, VCR, COL or VP16 in the presence of 3.3 μM and 6.6 μM VRP. No effects on cell growth were produced by VRP alone at these dose levels. It can be seen that the parent line is sensitised to all four drugs in the presence of 3.3 μM VRP and that a modest increase in SR is obtained by increasing the VRP dose to 6.6 μM. Although sensitisation of lines AR 1.0, VR 1.0 and CR 0.2 to ADM, COL and, to a lesser extent, VP 16 is obtained in the presence of 3.3 μM VRP it can be seen that, at this dose, the SRs for most MDR lines are generally less than, or no different from, those of the parent line. SRs greater than those observed for the parent line are seen in AR 1.0, VR 1.0 and CR 0.2 for VCR exposure only. The lowest SRs in the presence of 3.3 μM VRP are obtained consistently for CR 2.0.

It can be seen that, in the presence of 6.6 μM VRP, SRs for AR 1.0, VR 1.0 and CR 0.2 to ADM, VCR and COL are increased compared to those obtained at the lower VRP dose and generally exceed those obtained for the parent line. Whereas an increase in the SRs for CR 2.0 is observed with increased VRP dose, sensitisation of this line remains generally less than that obtained for the parent line. Sensitisation of all cell lines to VP 16 is only slightly increased at the higher dose of VRP.

*Chemosensitisation by cyclosporin A*

Table IV shows that the parent line is sensitised to all four drugs by 2.1 μM CsA and that a clear increase in SR at the higher dose of CsA is obtained for VCR and VP16 only. In general, at 2.1 μM CsA, SRs for MDR cell lines exceed markedly those obtained for the parent line, although only slight increases in SR are obtained for ADM at this dose. As observed for low dose VRP, the lowest SRs obtained in the MDR lines in the presence of low dose CsA are for CR 2.0. Further increases in SR are obtained at 4.2 μM CsA for all cell lines to all drugs. No effects on cell growth were produced by CsA alone at 2.1 μM but 20–30% growth inhibition was seen at 4.2 μM in EMT6/P, CR 0.2 and VR 1.0.

*Dose–response curves*

For EMT6/P, CR 0.2 and CR 2.0, detailed dose response data were obtained for VRP and CsA in combination with COL. Figure 5a shows a progressive increase in sensitisation of the three lines with increasing VRP dose. However, whereas only a small increase in SR is seen for the parent

**Table III** Effect of verapamil on the cytotoxic drug sensitivity of EMT6 sublines

| | *Sensitisation ratio*[a] | | | | | | | |
|---|---|---|---|---|---|---|---|---|
| | *ADM* | | *VCR* | | *COL* | | *VP16* | |
| *Cell* | *VRP* | | *VRP* | | *VRP* | | *VRP* | |
| *line* | *3.3 μM* | *6.6 μM* | *3.3 μM* | *6.6 μM* | *3.3 μM* | *6.6 μM* | *3.3 μM* | *6.6 μM* |
| EMT6/P | 4.3 | 8.2 | 1.1 | 2.2 | 2.6 | 3.0 | 1.6 | 1.8 |
| | 7.5 | 8.6 | 1.7 | 4.0 | 1.6 | 2.0 | 2.1 | 2.1 |
| | 3.8 | 8.0 | 1.8 | 3.5 | 2.0 | 2.8 | 1.6 | 2.1 |
| | 6.6 | | 1.7 | 5.6 | | | | |
| AR 1.0 | 3.0 | 6.0 | 2.4 | 6.5 | 2.6 | 4.4 | 1.2 | 1.0 |
| | 2.5 | 13.6 | 3.5 | 7.5 | 2.4 | 3.0 | 1.9 | 2.5 |
| | | | | | 3.5 | | | |
| | | | | | 1.5 | | | |
| VR 1.0 | 5.4 | 10.8 | 4.7 | 15.0 | 2.0 | 3.7 | 1.1 | 2.5 |
| | 4.1 | 15.3 | 3.2 | 7.2 | 2.3 | 3.7 | 1.4 | 2.3 |
| CR 0.2 | 5.8 | 11.7 | 2.7 | 4.4 | 2.8 | 5.8 | 1.8 | 2.1 |
| | 5.8 | 17.5 | 4.1 | 7.5 | 2.3 | 3.1 | 2.2 | 1.2 |
| CR 2.0 | 0.8 | 6.5 | 1.7 | 5.9 | 2.2 | 8.0 | 0.9 | 1.5 |
| | 0.8 | 2.3 | 1.4 | 3.5 | 1.0 | 1.8 | 1.1 | 1.0 |
| | 1.4 | | | | | 4.0 | | |

[a] $\text{Sensitisation ratio} = \frac{ID_{50} \text{ in absence of modifier}}{ID_{50} \text{ in presence of modifier}}$

Each value given represents the result of a separate experiment.

**Table IV** Effect of cyclosporin A on the cytotoxic drug sensitivity of EMT6 sublines

| | *Sensitisation ratio*[a] | | | | | | | |
|---|---|---|---|---|---|---|---|---|
| | *ADM* | | *VCR* | | *COL* | | *VP16* | |
| | *CsA* | | *CsA* | | *CsA* | | *CsA* | |
| *Cell line* | *2.1 μM* | *4.2 μM* | *2.1 μM* | *4.2 μM* | *2.1 μM* | *4.2 μM* | *2.1 μM* | *4.2 μM* |
| EMT6/P | 19 | 21 | 2.8 | 5.0 | 4.6 | 5.5 | 3.6 | 5.9 |
| | 22 | 16 | 4.8 | 8.3 | 5.1 | 6.0 | 3.8 | 6.3 |
| | 14 | 18 | 3.3 | 5.0 | 7.5 | 8.6 | 3.5 | 5.3 |
| | | | | | 8.2 | 10.4 | | |
| AR 1.0 | 29 | 95 | 18 | 54 | 61 | 118 | 13 | 29 |
| | 6.3 | 11 | 20 | 39 | 33 | 70 | 13 | 31 |
| | 17 | 73 | 5.5 | | 27 | 83 | | |
| VR 1.0 | 43 | 60 | 17 | 30 | 50 | 143 | 23 | 51 |
| | 23 | 50 | 54 | 88 | 47 | 70 | 23 | 78 |
| | 37 | 100 | 52 | 75 | 58 | 106 | | |
| CR 0.2 | 26 | 72 | 11 | 20 | 49 | 38 | 11 | 24 |
| | 15 | 19 | 57 | 80 | 23 | 48 | 19 | 20 |
| | 40 | 75 | 10 | 14 | 27 | 42 | | |
| | 11 | 32 | 9.2 | 11 | 25 | 36 | | |
| | 30 | 47 | | | 25 | | | |
| CR 2.0 | 24 | 72 | 6.0 | 25 | 12 | 180 | 7.8 | 30 |
| | 7.0 | 11 | 7.9 | 30 | 20 | 65 | 10 | 20 |
| | 35 | 72 | | | 15 | 104 | | |
| | 24 | 44 | | | 18 | 50 | | |

[a]See Table III.
Each value given represents the result of a separate experiment.

**Figure 5** Sensitisation ratios for cell lines EMT6/P (●), CR 0.2 (▲) and CR 2.0 (■) exposed to colchicine and either VRP (**a**) or CsA (**b**). Results shown are from a single experiment – a repeat experiment produced similar results.

line between 1.25 and 10 μM VRP, a much greater increase in SR is seen for the line CR 2.0 over this dose range.

Figure 5b shows dose–response data for CsA. It can be seen that for the parent line there is a gradual increase in SR with increasing CsA dose to 2.1 μM. No further increase in SR is seen at 4.2 μM. As seen for low dose VRP, doses of CsA between 0.26 and 1.0 μM give greater sensitisation of the parent line than the CR 2.0 cell line. In contrast, sensitisation of the CR 0.2 cell line is greater than that achieved in the EMT6/P line throughout the CsA dose range investigated. Although SRs for the CR 0.2 cell line increase steadily with increasing CsA dose, the shape of the curve in Figure 5b suggests that sensitisation may be approaching a plateau at 4.2 μM. In contrast, SRs for CR 2.0 rise sharply at 0.56 μM and continue to do so throughout the CsA dose range.

### Discussion

The EMT6 MDR variants described in the present study possess the biochemical and pharmacological characteristics typical of the MDR phenotype: cross-resistance to structurally and functionally unrelated cytotoxics, reduced cellular drug accumulation, hyperexpression of P-glycoprotein and partial reversal of drug resistance in the presence of the calcium channel blocker VRP. Selected variants also hyperexpress the cytosolic calcium binding protein sorcin/CP22, which is variably expressed in a number of MDR cell lines. Consistent with previous findings (Bradley *et al.*, 1988), the degree of drug resistance shown by the EMT6 MDR variants correlates well with the expression of P-glycoprotein only, in that CR 2.0 > AR 1.0 > VR 1.0 > CR 0.2 > EMT6/P for both P-glycoprotein and resistance. There is no apparent correlation with CP22 expression. This observation is in accordance with the concept that the gene for CP22 may be co-amplified with the P-glycoprotein gene as part of a single amplicon but is not a major determinant of drug resistance (van der Bliek *et al.*, 1986; Bradley *et al.*, 1988). In the present study, these MDR lines have been used to explore further the relationship between chemosensitisation and the expression of P-glycoprotein.

We have recently reported that MDR variants of the EMT6 tumour cell line show collateral cross-resistance to VRP, and that the degree of VRP resistance correlates with the amount of membrane P-glycoprotein (Reeve *et al.*, 1989). These data indicate that VRP is itself a substrate for the P-glycoprotein pump and are consistent with recent findings which show that VRP and cytotoxic drugs bind competitively to P-glycoprotein (Cornwell *et al.*, 1987; Safa *et al.*, 1987). Importantly, determination of the apparent dissociation constants for various agents revealed a range of cytotoxic drug and sensitiser affinities for P-glycoprotein (Naito & Tsuruo,

1989). Taken together, the aforementioned studies suggest that the ability of a reversing agent to overcome drug resistance to a particular cytotoxic in a given cell line is likely to be determined by the affinity of the reversing agent and the cytotoxic for P-glycoprotein and the amount of P-glycoprotein.

Support for this contention is provided by the findings of the present study. Comparison of the sensitisation efficacies of VRP and CsA shows clearly that CsA is more effective in overcoming MDR than VRP. Sensitisation of the MDR cell lines is generally greater than that of the parent line even at the lower dose of 2.1 μM and is consistently greater than that seen for VRP at 6.6 μM. Furthermore, in contrast to VRP, considerable sensitisation to VP16 is achieved with CsA. These findings are consistent with the observation that CsA has a much higher affinity for P-glycoprotein than VRP (Naito & Tsuruo, 1989) and thus support the contention that the affinity of a reversing agent for P-glycoprotein is indicative of its potency for overcoming drug resistance. Our findings show that higher doses of VRP than of CsA are generally required to produce SRs exceeding those seen in the parent line. It is important to note, however, that sensitisation to VCR at 3.3 μM VRP is greater than that seen in the parent for all MDR lines except CR 2.0. This observation is consistent with the finding that resistance to vinca alkaloids is more efficiently reversed by VRP than is resistance to ADM (Tsuruo *et al.*, 1983), an observation supported further by the finding that, in contrast to the other cytotoxics studied, a higher CsA dose is required in combination with ADM to produce SRs in the MDR lines greater than those seen in the parent line.

Our data also show that resistance to COL and VP16 is less effectively reversed by VRP than resistance to VCR. Both ADM and COL have lower affinities for P-glycoprotein than VCR (Naito & Tsuruo, 1989) and hence might be less effectively recognised and transported out of resistant cells by P-glycoprotein. Other mechanisms of resistance might therefore exist for these cytotoxic agents which result in a less complete reversal of resistance by VRP. However, the data show that CsA is able to overcome effectively resistance to COL and VP16, possibly indicating that this sensitiser may interact with multiple target molecules. Although both VRP and CsA can partially or completely reverse the drug accumulation defect seen in MDR cells (Nooter *et al.*, 1989; Coley *et al.*, 1989*b*), it is of course also entirely possible that these agents may each modify chemosensitivity by other mechanisms in addition to their interaction with P-glycoprotein.

The present study also shows the relationship between sensitiser dose, SR and the amount of P-glycoprotein expressed by a given cell line to be extremely complex. In general, the data shown in Tables III and IV do not indicate a clear relationship between the SR and the P-glycoprotein content for any of the cytotoxic drugs with either dose of either sensitiser. However, the more complete dose-response curves for EMT6/P, CR 0.2 and CR 2.0 exposed to COL in the presence of different doses of VRP or CsA do indicate a relationship between these parameters. In Figure 5a, it is only at the highest dose of VRP (10 μM) that sensitisation of CR 2.0 (high P-glycoprotein) exceeds that of EMT6/P and CR 0.2 (low P-glycoprotein). This trend is more clearly seen in Figure 5b, where sensitisation of CR 2.0 is actually less than that of EMT6/P at low doses of CsA, but the trend is reversed at higher doses. We believe that these data indicate that the small degree of resistance seen in cell lines with low P-glycoprotein can be optimally reversed by low sensitiser doses whereas much higher sensitiser doses are required to make a substantial impact on the high degrees of resistance seen in lines with high P-glycoprotein. If this is true, then it follows that the relative sensitisation measured in different cell lines using only a single sensitiser dose, as reported in many papers in the literature, is at best likely to be of limited value and, at worst, positively misleading.

Hence the findings of the present study indicate chemosensitisation to be a complex, multifactorial process depending on the cytotoxic drug under study, the sensitiser employed, sensitiser dose and the amount of P-glycoprotein in the target cell line. While some normal tissues have been shown to express high levels of P-glycoprotein, many normal cells contain low or undetectable amounts (Fojo *et al.*, 1987). A potentially important clinical consequence of our observations is that low doses of a reversing agent may sensitise relatively drug sensitive, low P-glycoprotein-containing, normal cells more effectively than drug-resistant tumour cells which hyperexpress P-glycoprotein.

### References

BECK, W.T., CIRTAIN, M.C., DANKS, M.K. & 5 others (1987). Pharmacological, molecular and cytogenetic analysis of 'atypical' multidrug-resistant human leukemic cells. *Cancer Res.*, **47**, 5455.

BRADLEY, G., JURANKA, P.F. & LING, V. (1988). Mechanism of multidrug resistance. *Biochim. Biophys. Acta*, **948**, 87.

CARMICHAEL, J., DE GRAFF, W.G., GAZDAR, A.F., MINNA, J.D. & MITCHELL, J.B. (1987). Evaluation of a tetrazolium-based semiautomated colorimetric assay: Assessment of chemosensitivity testing. *Cancer Res.*, **47**, 936.

COLE, S.P.C., DOWNES, H.F. & SLOVAK, M.L. (1989). Effect of calcium antagonists on the chemosensitivity of two multidrug-resistant human tumour cell lines which do not overexpress P-glycoprotein. *Br. J. Cancer*, **59**, 42.

COLEY, H.M., TWENTYMAN, P.R. & WORKMAN, P. (1989*a*). Identification of anthracyclines and related agents that retain preferential activity over Adriamycin in multidrug-resistant cell lines, and further resistance modification by verapamil and cyclosporin A. *Cancer Chemother. Pharmacol.*, **24**, 284.

COLEY, H.M., TWENTYMAN, P.R. & WORKMAN, P. (1989*b*). Improved cellular accumulation is characteristic of anthracyclines which retain high activity in multidrug resistant cell lines alone or in combination with verapamil or cyclosporin A. *Biochem. Pharmacol.*, **24**, 4467.

CORNWELL, M.M., PASTAN, I. & GOTTESMAN, M.M. (1987). Certain calcium channel blockers bind specifically to multidrug-resistant human KB carcinoma membrane vesicles and inhibit drug binding to P-glycoprotein. *J. Biol. Chem.*, **262**, 2166.

CROOP, J.M., GUILD, B.C., GROS, P. & HOUSMAN, D.E. (1987). Genetics of multidrug resistance: relationship of a cloned gene to the complete multidrug resistant phenotype. *Cancer Res.*, **47**, 5982.

DANO, K. (1973). Active outward transport of daunomycin in resistant Ehrlich ascites tumour cells. *Biochim. Biophys. Acta*, **323**, 466.

DEBENHAM, P.G., KARTNER, N., SIMINOVITCH, L., RIORDAN, J.R. & LING, V. (1982). DNA-mediated transfer of multidrug resistance and plasma membrane glycoprotein expression. *Mol. Cell Biol.*, **2**, 881.

FOJO, A.T., UEDA, K., SLAMON, D.J., POPLACK, D.G., GOTTESMAN, M.M. & PASTAN, I. (1987). Expression of a multidrug-resistance gene in human tumors and tissues. *Proc. Natl Acad. Sci. USA*, **84**, 265.

INABA, M., KOBAYASHI, H., SAKURAI, Y. & JOHNSON, R.K. (1979). Active efflux of daunorubicin and adriamycin in sensitive and resistant sublines of P388 leukemia. *Cancer Res.*, **39**, 2200.

KARTNER, N., EVERNDEN-PORELLE, D., BRADLEY, G. & LING, V. (1985). Detection of P-glycoprotein in multidrug resistant cell lines by monoclonal antibodies. *Nature*, **316**, 820.

KOCH, G.L.E., SMITH, M.J., MACER, D., WEBSTER, P. & MORTARA, R.A. (1986*a*). Endoplasmic reticulum contains a common abundant calcium-binding glycoprotein, endoplasmin. *J. Cell Sci.*, **86**, 217.

KOCH, G., SMITH, M., TWENTYMAN, P.R. & WRIGHT, K.A. (1986*b*). Identification of a novel calcium-binding protein (CP22) in multidrug resistant murine and hamster cells. *FEBS Lett.*, **195**, 275.

LAEMMLI, U.K. (1970). Cleavage of structural proteins during assembly of the head of bacteriophage T. *Nature*, **227**, 680.

MEYERS, M.B. & BIEDLER, J.L. (1981). Increased synthesis of a low molecular weight protein in vincristine-resistant cells. *Biochem. Biophys. Res. Commun.*, **99**, 228.

MOSMANN, T. (1983). Rapid colorimetric assay for cellular growth and survival: application to proliferation and cytotoxicity assays. *J. Immunol. Methods*, **65**, 55.

NAITO, M. & TSURUO, T. (1989). Competitive inhibition by verapamil of ATP-dependent high affinity vincristine binding to the plasma membrane of multidrug resistant K562 cells without calcium ion involvement. *Cancer Res.*, **49**, 1452.

NOOTER, K., OOSTRUM, R., JONKER, R., VAN DEKKEN, H., STOKDIJK, W. & VAN DER ENGH, G. (1989). Effect of cyclosporin A on daunorubicin accumulation in multidrug resistant P388 leukaemia cells measured by real-time flow cytometry. *Cancer Chemother. Pharmacol.*, **23**, 296.

RAMU, A., SPANIER, R., RAHAMINOFF, H. & FUKS, Z. (1984). Restoration of doxorubicin responsiveness in doxorubicin resistant P388 murine leukemia cells. *Br. J. Cancer*, **50**, 501.

REEVE, J.G., WRIGHT, K.A., RABBITTS, P.H., TWENTYMAN, P.R. & KOCH, G. (1989). Collateral resistance to verapamil in multidrug resistant mouse tumor cells. *J. Natl Cancer Inst.*, **81**, 1588.

RIORDAN, J.R. & LING, V. (1985). Genetic and biochemical characterisation of multidrug resistance. *Pharmacol. Ther.*, **28**, 51.

RIORDAN, J.R. & LING, V. (1979). Purification of P-glycoprotein from plasma membrane vesicles of Chinese hamster ovary cell mutants with reduced colchicine permeability. *J. Biol. Chem.*, **254**, 12701.

ROCKWELL, S.C., KALLMAN, R.F. & FAJARDO, L.F. (1972). Characteristics of a serially-transplanted mouse mammary tumor and its tissue-culture-adapted derivative. *J. Natl Cancer Inst.*, **49**, 735.

SAFA, A.R., GLOVER, C.J., SEWELL, J.L., MEYERS, M.B., BIEDLER, J.L. & FELSTED, R.L. (1987). Identification of the multidrug resistance-related membrane glycoprotein as an acceptor for calcium channel blockers. *J. Biol. Chem.*, **262**, 7884.

SLATER, L.M., SWEET, P., STUPECKY, M. & GUPTA, S. (1986*a*). Cyclosporin A reverses vincristine and daunorubicin resistance in acute lymphocytic leukaemia in vitro. *J. Clin. Invest.*, **77**, 1405.

SLATER, L.M., SWEET, P., STUPECKY, M., WETZEL, M.W. & GUPTA, S. (1986*b*). Cyclosporin A corrects daunorubicin resistance in Ehrlich ascites carcinoma. *Br. J. Cancer*, **54**, 235.

TOWBIN, H., STAEHELIN, T. & GORDON, J. (1979). Electrophoretic transfer of proteins from polyacrylamide gels to nitrocellulose sheet: procedure and some applications. *Proc. Natl Acad. Sci. USA*, **76**, 4350.

TSURUO, T., IIDA, H., TSUKAGOSHI, S. & SAKURAI, Y. (1982*a*). Increased accumulation of vincristine and adriamycin in drug-resistant tumour cells following incubation with calcium antagonists and calmodulin inhibitors. *Cancer Res.*, **42**, 4730.

TSURUO, T., IIDA, H., TSUKAGOSHI, S. & SAKURAI, Y. (1982*b*). Potentiation of vincristine and adriamycin effects in human hemopoietic tumor cell lines by calcium antagonists and calmodulin inhibitors. *Cancer Res.*, **43**, 2267.

TWENTYMAN, P.R. (1980). Response to chemotherapy of EMT6 spheroids as measured by growth delay and cell survival. *Br. J. Cancer*, **42**, 297.

TWENTYMAN, P.R. (1988). Modification of cytotoxic drug resistance by non-immuno-suppressive cyclosporins. *Br. J. Cancer*, **57**, 297.

TWENTYMAN, P.R., FOX, N.E. & BLEEHEN, N.M. (1986). Drug resistance in human lung cancer cell lines: cross-resistance studies and effects of the calcium transport blocker, verapamil. *Int. J. Radiat. Oncol. Biol. Phys.*, **12**, 1355.

TWENTYMAN, P.R., FOX, N.E. & WHITE, D.J.G. (1987). Cyclosporin A and its analogues as modifiers of adriamycin and vincristine resistance in a multidrug resistant human lung cancer cell line. *Br. J. Cancer*, **56**, 55.

TWENTYMAN, P.R. & LUSCOMBE, M. (1987). A study of some variables in a tetrazolium dye (MTT) based assay for cell growth and chemosensitivity. *Br. J. Cancer*, **56**, 279.

VAN DER BLIEK, A.M., VAN DER VELDE-KOERTS, T., LING, V. & BORST, P. (1986). Over expression and amplification of five genes in a multidrug resistant Chinese hamster ovary cell line. *Mol. Cell Biol.*, **6**, 1671.